# Green Extraction of Phenolic Compounds from Artichoke By-Products: Pilot-Scale Comparison of Ultrasound, Microwave, and Combined Methods with Pectinase Pre-Treatment

**DOI:** 10.3390/antiox14040423

**Published:** 2025-03-31

**Authors:** Lidia Gil-Martínez, José Manuel de la Torre-Ramírez, Sofía Martínez-López, Luis Miguel Ayuso-García, Giovanna Dellapina, Giovanna Poli, Vito Verardo, Ana María Gómez-Caravaca

**Affiliations:** 1 Department of Chemistry and Natural Products, DMC Research Center, Camino de Jayena s/n, 18620 Alhendín, Spain; lidiagm@dmcrc.com; 2 Environmental Department, National Technological Centre for the Food and Canning Industry (CTNC), C. Concordia, S/N, 30500 Murcia, Spain; sofiamartinez@ctnc.es (S.M.-L.); ayuso@ctnc.eu (L.M.A.-G.); 3 Experimental Station for the Food Preserving Industry—Research Foundation—Viale F. Tanara 31/a, 43121 Parma, Italy; giovanna.dellapina@ssica.it (G.D.); giovanna.poli@ssica.it (G.P.); 4 Institute of Nutrition and Food Technology ‘José Mataix’, Biomedical Research Center, University of Granada, Avda del Conocimiento sn., Armilla, 18100 Granada, Spain; vitoverardo@ugr.es (V.V.); anagomez@ugr.es (A.M.G.-C.); 5 Department of Nutrition and Food Science, University of Granada, Campus of Cartuja s/n, 18071 Granada, Spain; 6 Department of Analytical Chemistry, University of Granada, Campus of Fuentenueva s/n, 18071 Granada, Spain

**Keywords:** circular economy, pectinase pretreatment, natural extracts, UAE, MAE, artichoke by-products

## Abstract

The revalorization of artichoke (*Cynara scolymus* L.) by-products is a promising strategy to obtain bioactive compounds with antioxidant properties, supporting a circular economy approach. This study compares the efficiency of an enzymatic pretreatment followed by microwave-assisted extraction (EMAE), ultrasound-assisted extraction (EUAE), and ultrasound-microwave-assisted extraction (EUMAE) at a pilot scale for recovering antioxidant compounds. Extracts were purified using Diaion^®^ HP20 resin to obtain phenolic-rich fractions with enhanced antioxidant activity. The results showed that EUAE was the most effective technique, achieving a total phenolic content (TPC) of 210.76 ± 1.40 µmol GAE/g d.w. with an extraction yield of 21.38%. HPLC-MS analysis identified 14 major phenolic compounds, including chlorogenic acid isomers (60.73 mg/g d.e.), caffeic acid (34.29 mg/g d.e.), and luteolin rutinoside (103.27 mg/g d.e.), among others, which contribute to the extracts’ high bioactivity. The antioxidant potential of the extracts was assessed using Folin–Ciocalteu (F-C), ABTS, DPPH, and FRAP assays. EUAE extracts exhibited the highest antioxidant activity values, with F-C: 985.33 ± 4.46 µmol GAE/g d.e., ABTS: 80.46 ± 2.39 µmol TE/g d.e., DPPH: 87.03 ± 1.11 µmol TE/g d.e., and FRAP: 184.99 ± 2.52 µmol TE/g d.e. The purification process using Diaion^®^ HP20 resin further enhanced TPC and antioxidant activity, with the enzyme–ultrasound-assisted extraction—purified extract (EUAE-PE) reaching a phenolic purity of 50.71% and an F-C value of 2981.35 ± 12.16 µmol GAE/g d.e.

## 1. Introduction

The artichoke (*Cynara scolymus* L.) is a perennial plant from the *Asteraceae* family, extensively cultivated in countries such as Italy, Spain, and Egypt. As a key component of the Mediterranean diet, it is widely consumed in various forms, including fresh, canned, roasted, and baked [1,2]. As a recognized functional food, artichokes exhibit antioxidant, hepatoprotective, anti-inflammatory, hypoglycemic, and antimicrobial properties [3,4,5,6,7]. Furthermore, artichoke leaf extracts have been traditionally used in herbal medicine for liver and gallbladder disorders, and they are commercially available for their hepatoprotective and lipid-lowering effects [3,8]. While the edible portion consists of the receptacle of immature flowers and inner bracts, up to 85% of the plant’s biomass—primarily leaves, stems, and roots—is discarded as waste. It is estimated that industrial processing alone generates approximately 800 tons of residues annually from fresh-cut production [9]. Moreover, due to their high moisture content, these by-products are highly susceptible to microbial growth, raising environmental concerns [5]. Nevertheless, artichoke by-products represent a promising source of valuable bioactive compounds, including phenolics, flavones, terpenes, inulin, and dietary fiber, which makes them suitable for use in the food, nutraceutical, and pharmaceutical sectors. After recovering these high-value components, the remaining solid material can be further utilized for energy production, as a nutrient source in fermentation or anaerobic digestion processes, or through methods such as composting, pyrolysis, or use as animal feed. These strategies support a sustainable food system and encourage the adoption of circular economy principles in industrial processing, fostering a shift toward bio-based production models [5,10,11,12,13,14]. The selection of efficient extraction methods plays a key role in improving the economic viability and competitiveness of industries that rely on phytochemicals. Optimizing these processes not only helps reduce production costs but also maximizes the recovery of bioactive compounds from plant matrices and improves the quality and purity of the final extracts, which are critical attributes for their incorporation into pharmaceutical, nutraceutical, and cosmetic formulations [15].

Conventional extraction techniques, often reliant on chemical solvents, typically require large volumes of aggressive reagents that can leave undesirable residues. Additionally, they tend to involve elevated temperatures and prolonged durations, resulting in high energy consumption [16]. On the other hand, green extraction approaches aim to reduce environmental impact by enhancing safety for operators, minimizing energy use, improving waste management, and substituting hazardous chemicals with environmentally friendly alternatives whenever possible [17]. Compared to conventional techniques, these green methods are capable of disrupting the rigid plant cell wall structures more effectively, achieving higher extraction yields in shorter processing times, and eliminating the need for excessive heating or toxic solvents. As a result, the obtained extracts maintain their bioactivity and can be marketed under a clean and sustainable label [16].

Recent research has focused on optimizing extraction techniques for bioactive compounds from artichoke waste, including maceration [18], microwave-assisted extraction (MAE) [14,19], ultrasound-assisted extraction (UAE) [20,21,22], pressurized liquid extraction (PLE) [12,23,24], pulsed electric field (PEF) [7,25] and natural deep eutectic solvents (NADES) [26]. However, no research has been conducted on the efficiency of the combination of extraction techniques such as enzyme-assisted, ultrasound, and microwaves on the bioactive compounds recovery from artichoke by-products. In this context, sequential extraction strategies—based on a two-step approach—have shown greater effectiveness in extracting higher levels of polyphenols and more active flavonoids when compared to traditional single-step methods [27]. However, scaling up these methods for industrial applications remains a challenge.

Additionally, it is widely recognized that plant-based extracts contain a complex mixture of compounds and impurities, including carbohydrates, amino acids, peptides, and lipid-derivative substances, which can interfere with or diminish their pharmacological efficacy [28]. Although several purification methods, such as solvent fractionation, membrane separation, and preparative HPLC, can be used, their high cost and limited scalability hinder their use for industrial applications [28,29]. In contrast, resin adsorption has been employed to purify phenolic compounds at a pilot scale. This method has demonstrated high efficiency in selectively concentrating polyphenols and is currently being adopted at the industrial level thanks to its cost-effectiveness, reduced solvent use, operational simplicity, and ability to produce residue-free extracts [30,31,32].

This study aims to bridge the gap between laboratory-scale studies and industrial applications by evaluating, at a pilot scale, the effectiveness of enzymatic pretreatment combined with microwave-assisted extraction (EMAE), ultrasound-assisted extraction (EUAE), and a hybrid ultrasound-microwave assisted extraction (EUMAE for the recovery of antioxidant compounds from artichoke waste. Additionally, the study includes a purification stage using Diaion^®^ HP20 resin to concentrate phenolic compounds and obtain extracts with an enhanced antioxidant capacity. Moreover, the main bioactive constituents present in the extracts were tentatively identified through HPLC-MS analysis. Notably, this is the first study to employ a pilot-scale ultrasound–microwave integrated system to extract phenolic compounds using green solvents from the biomass of artichoke by-products.

## 2. Materials and Methods

### 2.1. Chemicals and Reagents

Reagents including 2,2-diphenyl-1-picrylhydrazyl (DPPH), 2,2′-azino-bis (3-ethylbenzothiazoline-6-sulfonic acid) (ABTS), 2,4,6-tripyridyl-S-triazine (TPTZ), potassium persulfate, Trolox, gallic acid (≥98%), and the Folin–Ciocalteu reagent, along with various reference compounds, were purchased from Sigma-Aldrich (St. Louis, MO, USA). Merck KGaA (Darmstadt, Germany) supplied glacial acetic acid, iron chloride hexahydrate, sodium carbonate, and LC-MS-grade methanol and water. Additional standards, including rutin (≥95%), catechin (≥99%), caffeic acid (≥99%), and apigenin (≥98%), were also obtained from Sigma-Aldrich (St. Louis, MO, USA). Fungal pectinase enzyme (Altaquímica, Barcelona, España) was used for the enzymatic pre-treatment. Denatured ethanol supplied by Arquimi (Massanassa, Valencia, Spain) and tap water were used as solvents for the pilot extraction.

### 2.2. Plant Material

A 180 kg amount of fresh artichoke by-products (leaves and stems) was kindly provided by the association of producers-exporters of fruits and vegetables Proexport (Murcia). They were washed and air dried in a chamber at 40 °C until constant weight. Then, they were grounded in a pilot cutter (Mainca, Granollers, Spain) at 3000 rpm for 5 min. The grounded material was separated in 4 kg batches under vacuum at −20 °C until use in the pilot run.

### 2.3. Pilot Scale Processes

#### 2.3.1. Equipment

A reactor with a volume of 120 L, equipped with a variable-speed mechanical stirrer and with a double-walled system for temperature regulation of the processes that integrate microwave and ultrasound technologies, was used for the heating and extraction processes (Sairem, Lyon, France). The microwave power can be adjusted within a range of 600–6000 W at 2450 MHz, while the ultrasonic power varies from 200 W to 1800 W at 20 kHz.

#### 2.3.2. Enzyme-Assisted Extraction Pre-Treatment (EAE)

The water (12 L) was preheated at 50 °C in the reactor. Then, 4 kg of chopped dry artichoke wastes were added to complete a solid-to-liquid (S/L) ratio of 1:3. Then, 60 mL of enzyme were added to the mixture, and the temperature was maintained for 3 h at 50 °C, stirring at 30 rpm. An aliquot of 50 mL was centrifuged, and the aqueous phase vacuum vacuum-dried for subsequent analysis after the incubation period for enzymatic pretreatment. Then, 24 L of ethanol and additional 4 L of water were added to reach a 60:40 ethanol-to-water ratio and a 1:10 S/L ratio. This procedure was consistently applied across all experimental assays, followed by the implementation of the different extraction processes.

#### 2.3.3. Microwave-Assisted Extraction (EMAE)

EMAE was performed at a power setting of 5000 W for 7 min reaching a temperature of 60 °C under continuous stirring at 30 rpm. The total extraction process lasted 35 min. Upon completion, the separation of the solid organic phase and the extract was carried out by filtration through a 50-micron mesh. An aliquot of 1 L of the extract was centrifuged, and the supernatant evaporated to dryness using a vacuum rotary evaporator, the weight of the dry mass was recorded, and it was stored at −20 °C until further analysis.

#### 2.3.4. Ultrasound-Assisted Extraction (EUAE)

The ultrasound-assisted extraction was performed by applying a power of 1200 W for 1 h under continuous stirring at 30 rpm reaching a maximum temperature of 30 °C. Once the extraction was completed, the separation of the solid organic phase and the extract was carried out by filtration using a 50-micron mesh. A 1 L aliquot of the extract was centrifuged and the supernatant was concentrated to dryness using a vacuum rotary evaporator, with the dry mass weight recorded before being stored at −20 °C for further analysis.

#### 2.3.5. Ultrasound–Microwave-Assisted Extraction (EUMAE)

For the integrated microwave- and ultrasound-assisted extraction, a power of 5000 W was applied using the microwave until a temperature of 60 °C was reached, maintaining the stirring at 30 rpm for 7 min while ultrasound was employed at 1200 W power simultaneously. The entire process lasted 35 min. Once the extraction was completed, the organic phase was separated by filtration with a 50-micron mesh. A 1 L portion of the extract was centrifuged and the supernatant dried to completion using a vacuum rotary evaporator, the dry mass was weighed and then stored at −20 °C for subsequent analysis.

#### 2.3.6. Process of Phenolic Compounds Purification

The phenolic fraction purification process was carried out using the methodology described by Ramalakshmi et al., with some modifications [33]. For this, Diaion^®^ HP20 resin, composed of highly porous styrene–divinylbenzene copolymer was used. The procedure involved an initial conditioning of the raw ethanolic extracts and the resin. First, solvent was removed from an aliquot of 500 mL of the extracts through vacuum rotary evaporation. The extracts were reconstituted in 300 mL of distilled water in order to obtain aqueous extracts to be used for the purification. Then, the resin was conditioned in ethanol for 30 min. Subsequently, ethanol was discarded by filtration, and the resin was thoroughly washed with distilled water to ensure complete solvent elimination. The resin was then incubated with the aqueous extract at a 5% (*w*/*v*) ratio of resin to extract volume for 3 h at room temperature under orbital agitation. After this incubation period, the supernatant was filtrated and discarded, and the resin was washed with water until complete removal of sugars (confirmed using a refractometer at 0° Brix) and acids (neutral pH). Polyphenol desorption was then carried out by incubating the resin with ethanol for one hour in agitation. This process was repeated twice. Finally, the obtained supernatant was concentrated by vacuum rotary evaporation, yielding the purified polyphenol extract. The purified polyphenol extracts were designated as EMAE-PE, EUAE-PE, and EUMAE-PE.

### 2.4. Determination of the Extraction Yield

The extraction yield, expressed as the grams of dry extract obtained from 100 g of dry artichoke by-product was calculated using the following formula:

(1) $$Extraction\;yield\;\left(\%\right)=\frac{\;Dry\;Extract\;(g)}{Dry\;Artichoke\;byproduct,\;(g)}\times 100$$

### 2.5. Determination and Quantification of Bioactive Compounds by HPLC-MS

The characterization and quantification of active compounds in artichoke by-products were performed using an Agilent 1200 HPLC system coupled to a QTOF Agilent 6520 B mass spectrometer with an InfinityLab Poroshell 120 EC-C18 column (2.1 mm × 50 mm, 2.7 μm). The HPLC method used a 0.35 mL/min flow rate with a gradient of water (0.1% formic acid) (A) and acetonitrile (B): from 4% to 100% B over 25.5 min, held until 31 min, then re-equilibrated in 7.5 min.

Mass spectrometry analysis was performed in negative electrospray ionization (ESI) mode, with a capillary voltage of 3500 V, nebulizer pressure of 50 psi, gas flow of 10 L/min at 365 °C, and Auto MS/MS mode (50–1200 *m*/*z*) with two precursors per cycle, 25 V collision energy, and N_2_ as the collision gas. Data processing was carried out using Agilent MassHunter Software (B.06.00).

### 2.6. Determination of Antioxidant Activity

#### 2.6.1. Folin–Ciocalteu (F-C) Assay

The total phenolic content (TPC) or total antioxidant content (TAC) of the extracts was evaluated using the F–C method, adapted from Ainsworth et al. with minor modifications [34]. In brief, 400 μL of either the sample, a standard solution, or an 80% methanol blank was mixed with 800 μL of a 10% (*v*/*v*) F–C reagent in test tubes. Next, 3200 μL of a 700 mM Na_2_CO_3_ solution was added, and the mixture was left to incubate at room temperature for two hours. Absorbance was recorded at 765 nm. Gallic acid, prepared in concentrations ranging from 25 to 400 ppm, was used as the reference standard. Results were expressed as micromoles of gallic acid equivalents per gram of dry artichoke wastes (μmol GAE/g d.w.) or micromoles of gallic acid equivalents per gram of dry extract (μmol GAE/g d.e.). Assays were carried out in triplicate.

#### 2.6.2. Trolox Equivalent Antioxidant Capacity (TEAC) Assay

The TEAC assay was conducted according to the methodology described by Re et al. [35]. The ABTS^·^⁺ radical cations were produced by reacting a 7 mM ABTS solution with 2.45 mM potassium persulfate, allowing the reaction to proceed for 16 h. The resulting solution, with an absorbance of 734 nm, was then adjusted to a final absorbance of 1.1 (±0.02). For the assay, 2850 μL of the ABTS^·^⁺ solution was mixed with 150 μL of either the blank (solvent), a standard, or the sample. The results were reported as the mean ± standard deviation (SD) of three replicates in μmol Trolox equivalents per gram of dry extract (μmol eq. Trolox/g d.w.).

#### 2.6.3. Ferric Reducing Antioxidant Power (FRAP) Assay

The FRAP assay was carried out following the protocol by Benzie and Strain, with slight modifications [36]. The FRAP reagent was prepared by mixing 10 parts of a 300 mM acetate buffer (pH 3.6), 1 part of a 10 mM TPTZ solution in acid, and 1 part of a 20 mM FeCl_3_ solution. Trolox was used as the standard for the analysis. In triplicate, 3000 μL of the FRAP reagent was combined with 480 μL of either the blank (solvent), a standard, or the sample, and the absorbance was measured at 593 nm. The results were expressed in micromoles of Trolox equivalents per gram of dry extract (μmol eq. Trolox/g d.w.).

#### 2.6.4. DPPH Assay

The antioxidant activity of the extract was determined using a modified version of the DPPH radical scavenging assay described by Brand-Williams [37]. In summary, 100 μL of either the solvent (blank) or sample was mixed with 3900 μL of a 60 μM DPPH solution, in triplicate. The reaction took place in darkness at room temperature for 30 min, and absorbance was recorded at 515 nm. Trolox was used as standard. The results were expressed in micromoles of Trolox equivalents per gram of dry extract (μmol eq. Trolox/g d.w.).

### 2.7. Statistical Analysis

A one-way analysis of variance (ANOVA) test, supplemented with Tukey’s post hoc test, was used for the statistical comparison of the results. Differences were considered statistically significant when *p* < 0.05. The analyses were conducted using the Statistica 7.0 software package (StatSoft, Tulsa, OK, USA). All results were presented as mean ± standard deviation (SD).

## 3. Results and Discussion

### 3.1. Determination of Yield of Extraction and Total Phenolic Content of the Extracts

#### 3.1.1. Enzyme-Assisted Extraction (EAE) Pretreatment

Among the innovative environmentally friendly techniques for extracting bioactive compounds, enzyme-assisted extraction (EAE) has gained significant attention. This method enhances process efficiency by allowing subsequent extractions to be carried out at lower temperatures, reducing energy consumption and preserving thermosensitive compounds [38]. Pectinases are naturally occurring enzymes found in higher plants and microorganisms, including Aspergillus niger, from which they were obtained in this study. These enzymes catalyze the hydrolysis of pectin, a polysaccharide that accounts for approximately 5–12% of the plant cell wall and plays a crucial role in maintaining its structural integrity by interconnecting cellulose and hemicellulose fibers. Other key cell wall components include hemicelluloses, cellulose, arabinans, and arabinogalactans. By breaking down pectin, pectinases weaken the cell wall, increasing its permeability and facilitating the release of intracellular bioactive compounds such as polyphenols and flavonoids [39,40]. This enzyme was selected based on its superior efficiency in extracting phenolic compounds, as observed in previous laboratory-scale experiments. These preliminary tests demonstrated that pectinolytic enzymes were more effective than hemicellulases in solubilizing bioactive compounds into the aqueous medium in artichoke wastes (unpublished data). These findings align with those reported by Thang et al., who compared the efficiency of pectinase, cholesterol esterase, and cellulase for extracting cynarine and chlorogenic acid from dried artichoke leaves. Their results confirmed that pectinase was the most effective enzyme for phenolic extraction from artichoke leaf biomass [41].

To monitor the extraction of bioactive compounds, the extraction yield and total phenolic content (TPC) in the dried aqueous phase were analyzed and calculated. The results, presented in Table 1, indicate an extraction yield of 14.87% and a TPC of 44.82 μmol GAE/g d.w. or expressed in another way in order to compare with the literature 301.40 ± 2.07 μmol GAE/g d.e. In a study conducted by Ayuso et al., an aqueous extract with a TPC of 752.4 μmol GAE/g d.e. was obtained using Viscozyme^®^ L as the enzymatic treatment, with artichoke external bracts as a by-product [42]. In this case, a higher recovery of phenolic compounds was achieved. However, comparing the efficiency of two processes using different raw materials is challenging due to biological and agronomic factors that influence the total phenolic content of the raw biomass used for the extractions. It is well established that phenolic compounds are not uniformly distributed throughout the artichoke plant and that the plant genotype, along with environmental and agronomic growth conditions, influences the total phenolic content in artichoke [43,44,45]. Then, it is impossible to compare the efficiency of the extraction treatments if the TPC of the raw biomass is unknown.

#### 3.1.2. Extraction of Phenolic Compounds by MAE, UAE and UMAE

Extraction yields of phenolic compounds used to be lower in aqueous extractions compared to those carried out with ethanol–water mixtures as a solvent, as ethanol enhances the extraction of phenolic compounds by improving their solubility due to its intermediate polarity, disrupting cell wall interactions, increasing membrane permeability and denaturing polyphenol oxidases [1]. Thus, in the present study, the addition of ethanol to the mixture and the application of microwaves (EMAE), ultrasounds (EUAE), and the combination of microwaves and ultrasounds (EUMAE) was carried out to enhance antioxidant recovery. Results are presented in Table 2.

The extraction yield and TPC results confirm that using ethanol and microwaves or ultrasounds improves the recovery of antioxidant compounds from the raw materials when compared with enzymatic treatment alone. Furthermore, EUMAE achieves the highest yield of extraction (24.68 ± 2.74%) surpassing individual EMAE (22.13 ± 1.34%) and EUAE (21.38 ± 0.95%), which do not present differences between them. However, although the highest TPC was also obtained with EUMAE (211.35 ± 3.12 µmol GAE/g d.w.), there were no significant differences between total phenolic recovery using EUAE or EUMAE. These results highlight EUAE as the most effective method for phenolic extraction, being more selective than EUMAE. While EUMAE may be advantageous when prioritizing total extractable matter, it has not demonstrated clear benefits over EUAE in terms of TPC, and EMAE has proved to be the less efficient extracting methodology for phenolic compound recovery from artichoke wastes in our study.

The results obtained are in accordance with the results obtained by Rodsamran et al., which proved UAE to be more efficient than MAE in the extraction of phenolic compounds from lime peel wastes [46]. In contrast, Awad et al. compared MAE and UAE in the extraction of phenolic compounds from artichoke by-products obtaining better results for MAE than UAE [47].

The absence of prior studies utilizing ultrasound–microwave-assisted extraction (UMAE) for the recovery of bioactive compounds from artichoke, makes it difficult to compare results with other similar studies. However, our results are consistent with those reported for other matrices. For instance, Bich et al. found that UMAE was less effective than UAE in the extraction of phenolic compounds and antioxidants from mangosteen [48]. Similarly, Jiang et al. observed that UAE and MAE alone were more effective than UMAE in recovering phenolic compounds from fig leaves [49]. Despite this, the increase in total extraction yield observed with UMAE suggests a more efficient mass transfer process, which could favor the recovery of other bioactive compounds such as fibers and proteins.

Comparing our findings with previous studies on artichoke by-products further underscores the efficiency of EUAE and EUMAE. While 180 μmol GAE/g d.w. were recovered from artichoke leaves and stems using EMAE, Mena-García et al. reported TPC values of 49 μmol GAE/g d.w. for artichoke stalks, 113 μmol GAE/g d.w. for leaves, and 194 μmol GAE/g d.w. for bracts using MAE under controlled conditions (98 °C, 3 min, 50% ethanol) [14]. Kayahan et al. obtained a TPC recovery of 35.7 μmol GAE/g d.w from artichoke bracts and leaves using an optimized MAE protocol (6 min of extraction, 50% ethanol, and 80 °C) [19]. With regard to UAE, we achieved a recovery of 210 μmol GAE/g d.w., a quantity superior to that described in other published research. For example, Cannas et al. optimized the extraction of bioactive compounds from stems, bracts, and leaves of artichokes using UAE. They found differences in the optimal times and ethanol–water ratios between stems (42% ethanol, 10 min of extraction), leaves (20% ethanol, 10 min), and bracts (64% and 41 min), with TPC results of 148 μmol GAE/g d.w for stems, 101 μmol GAE/g d.w. for leaves and 118 μmol GAE/g d.w. for bracts, respectively [22]. Cioni et al. used a similar methodology for the extraction of phenolic compounds from artichoke wastes. They started with an enzymatic pretreatment using pectinase and β-glucosidase, added ethanol to denature the enzymes and assist the extraction of phenolic compounds, and followed with a UAE treatment for 15 min achieving a TPC recovery of 18.3 μmol GAE/g d.w. Therefore, although at first glance the results obtained in our study appear to be superior to those reported in other works, we must be cautious when making comparisons. As previously mentioned, the extraction yield of phenolic compounds does not solely depend on the technology employed or the extraction conditions used. Instead, the final results are primarily influenced by the phenolic compound content present in the starting plant material.

### 3.2. Purification of Phenolic Compounds

**Figure 1:**
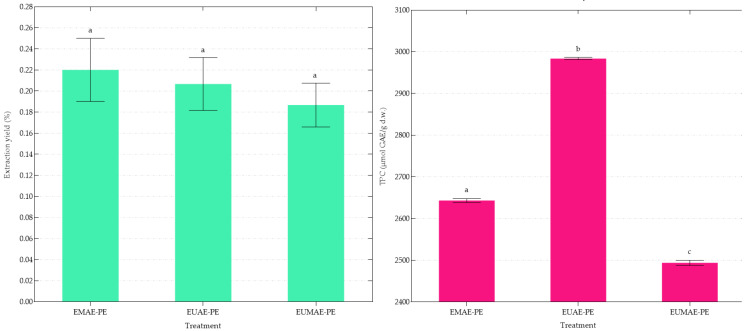
Left: Results of extraction yield, as a % of g of dry extract obtained from 100 g of dry artichoke (±SD) (n = 3). Right: Results of TPC as μmol GAE per g of dry artichoke (±SD) (n = 3). Results with different letters are significantly different (*p* < 0.05).

The purification of phenolic compounds using the Diaion^®^ HP20 resin was evaluated by analyzing the extraction yield and TPC across the different extraction methods as shown in Figure 1.

The extraction yields ranged from 0.19% to 0.22%, with enzyme–microwave-assisted extraction-purified extract (EMAE-PE) exhibiting the highest yield. The relatively low yields obtained may be attributed to several factors. First, the phenolic content in artichoke by-products is generally lower than in edible parts [43]. Additionally, the purification step, including the adsorption–desorption process on the resin, also plays a critical role because the incomplete adsorption of certain phenolics or the inefficient desorption during elution may contribute to compound loss [29]. However, the small and non-significant differences observed between the methods tested suggest that the extraction mechanism was not significantly influenced by the type of energy applied. In terms of TPC, enzyme–ultrasound-assisted extraction-purified extract (EUAE-PE) demonstrated the highest concentration of phenolic compounds (2983.65 ± 5.62 µmol GAE/g d.e.), surpassing EMAE-PE (2643.21 ± 9.48 µmol GAE/g d.e.) and enzyme–ultrasound–microwave-assisted extraction-purified extract (EUMAE-PE) (2493.87 ± 12.31 µmol GAE/g d.e.). These results suggest that the EUAE-PE extract was obtained from an extract with a higher content of phenolic compounds (EUAE) compared to those obtained through EMAE and EUMAE. This difference appears to become more discernible following the purification process, during which interfering substances such as organic acids or amino acids (potentially reacting with the F-C reagent and leading to overestimated TPC values) are removed [50]. At the end of the process, we produced artichoke by-product extracts with a polyphenol content of 44.93% using EMAE-PE, 50.71% using EUAE-PE, and 42.39% using EUMAE-PE. The superior performance of EUAE-PE suggests that EUAE enhances the recovery of phenolic compounds or that it increases the recovery of molecules with high antioxidant potential, that might be more thermolabile and susceptible to degrade using EMAE or EUMAE.

The purification process appears to be efficient in selectively retaining phenolic compounds, as indicated by the relatively high TPC values post-purification and in accordance with other studies previously published. For example, Ren et al. developed a purified phenolic extract from wampee fruit by-products with a purity of 53.8% using AB-8 macroporous resin [51]. Conidi et al. achieved a successful recovery of chlorogenic acid and apigenin glucoside from artichoke wastewaters using membrane filtration and Lewatit crosslinked polystyrene resins [52]. Moreover, Gore et al. prepared an enriched phenolic fraction from seabuckthorn using Diaion^®^ HP20 resin with a purity of 29.1% [53]. Thus, although the use of resins significantly reduces extraction yields, it enables the production of highly concentrated extracts that could be of great interest to the cosmetic or pharmaceutical industry.

### 3.3. Determination and Quantification of Bioactive Compounds by HPLC-MS

**Figure 2:**
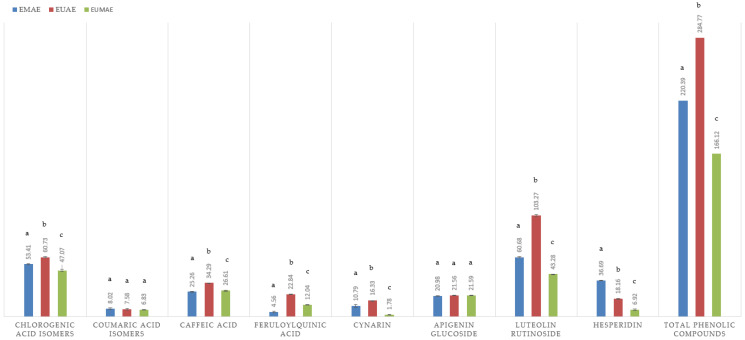
Detailed quantification of the phenolic compounds identified and obtained through different extraction methods: EMAE (blue), EUAE (red), and EUMAE (green). Results are expressed as the mean (n = 3) (±SD). The statistical differences between the extraction methods for each compound are indicated by distinct letters (*p* < 0.05).

The phenolic compounds present in the extracts were tentatively characterized and quantified using HPLC-MS, with compound identification based on mass spectral data comparison with databases, commercial standards (when available), and the literature reports. The molecular formula and the agreement between experimental and calculated *m*/*z* values further supported the tentative assignments. Results are summarized in Table 3 and Figure 2.

As it can be observed, 13 different molecules could be identified. A total of eight phenolic acids and derivatives were identified, all of them previously described in artichoke. Three different isomers, eluting at retention times (Rt) 2.78, 6.017, and 6.386 min, could be tentatively identified thanks to their *m*/*z* 353 Da and molecular formula C_16_H_18_O_9_ as chlorogenic acid isomers. Chlorogenic acid has been widely identified and is a characteristic antioxidant compound present in artichoke plants and extracts [3,54,55]. It consists of a quinic acid molecule conjugated with a caffeic acid molecule and exhibits various nutraceutical properties, such as antioxidant, antibacterial, anti-obesity, antitumor, and anti-inflammatory activities [56].

Moreover, two coumaric acid isomers were presumptively identified at Rt = 5.214 min and Rt = 7.203 min, thanks to its *m*/*z* and molecular formula C_9_H_8_O_3_ [20,57]. Coumaric acid exhibits antioxidant properties thanks to the presence of its phenyl hydroxyl group. It also exerts mild antimicrobial and anticancer activities, and similarly to other phenolic compounds, it can reduce the intestinal absorption of carbohydrates and modulate enzymes involved in glucose metabolism exerting a positive effect in diabetes mitigation [58]. Additionally, caffeic acid (C_9_H_8_O_4_) was detected at Rt = 5.214 min. It was confirmed by comparison of the mass spectra and Rt with the standard [3,54]. Caffeic acid has demonstrated antioxidant, anti-inflammatory, and anticancer properties both, in vitro and in vivo. The anticancer activity of caffeic acid is associated with its dual role as an antioxidant and pro-oxidant, which is influenced by its chemical structure, particularly with the presence, number, and position of free phenolic hydroxyl groups in the catechol moiety, as well as the double bond in the carbon chain [59].

Moreover, in peak 10, a compound with *m*/*z* 367 and molecular formula C_17_H_20_O_9_ was putatively characterized as feruloylquinic acid, which has demonstrated that may be involved in protection processes against hepatotoxicity and antioxidant and anticancer bioactivities [24,55,60]. Cynarin, with *m*/*z* 515 and a molecular formula C_25_H_24_O_12_, was also tentatively identified at Rt = 12.223 min [12,24]. It is also known as 1,3-dicaffeoylquinic acid and thanks to its molecular structure, it exhibits potent antioxidant, hepatoprotective, and anti-inflammatory properties, contributing to its potential health benefits and therapeutic applications [61].

Moreover, three flavonoids and derivatives were tentatively identified. At Rt = 5.81 min, apigenin glucoside, with *m*/*z* 431 and molecular formula C_21_H_20_O_10_ [12,54]. Peak 11, with *m*/*z* 593 and molecular formula C_27_H_30_O_15,_ was tentatively identified as luteolin rutinoside [55], and peak 12, with *m*/*z* 609 and molecular formula C_28_H_34_O_15_ was tentatively identified as hesperidin [4]. All these phenolic compounds identified may be associated with various health benefits associated with artichoke consumption, including anti-inflammatory effects, liver protection, cholesterol-lowering properties, and improved digestive health [62,63,64,65].

The extraction efficiency of phenolic compounds varied significantly depending on the method employed. In particular, chlorogenic acid isomers exhibited the highest concentration in the EUAE samples (60.73 mg/g d.e.), with EMAE yielding slightly lower amounts (53.41 mg/g d.e.), whereas EUMAE resulted in the lowest recovery (47.07 mg/mL). This suggests that enzyme–ultrasound-assisted extraction (EUAE) enhances the solubilization of these compounds.

**Figure 3:**
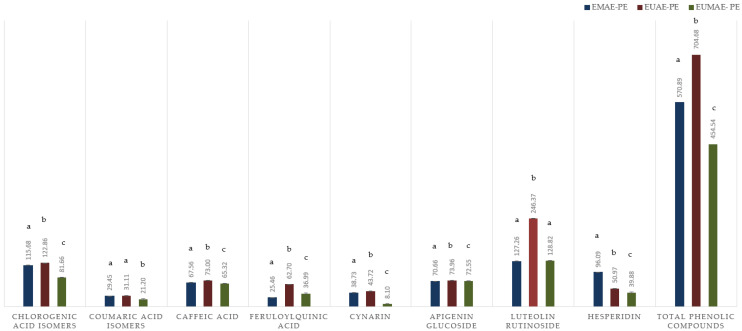
Detailed quantification of the phenolic compounds identified and obtained in the concentrated phenolic extracts after resin purification: EMAE-PE (dark blue), EUAE-PE (dark red), and EUMAE-PE (dark green). Results are expressed as the mean (n = 3) (±SD). Statistical differences between the extracts for each compound are indicated by different letters (*p* < 0.05).

Results of comprehensive phenolic compound quantification in raw and purified artichoke by-product extracts are presented in Figure 2 and Figure 3, respectively.

For coumaric acid isomers, all three methods yielded relatively low amounts with no significant differences among them. Caffeic acid followed a similar trend to feruloylquinic acid, with EUAE achieving the highest recovery (34.29 mg/g d.e.) and 22.84 mg/g d.e., respectively. In this case, the amount recovered by EUMAE (26.61 mg/g d.e.) for caffeic acid and 12.04 for feruloylquinic acid was slightly higher than EUMAE, showing the lowest extraction efficiency. Regarding cynarin, EUAE again provided the highest extraction yield (16.33 mg/g d.e.), followed by EMAE and EUMAE.

Conversely, apigenin glucoside did not show significant differences among the extraction techniques, suggesting that this flavonoid is equally extractable under all tested conditions. For flavonoids such as luteolin rutinoside, a remarkable difference in the recoveries was observed. EUAE resulted in an exceptionally high extraction yield (103.27 mg/g d.e.), significantly surpassing both EMAE (60.68 mg/g d.e.) and EUMAE (43.28 mg/g d.e.).

Lastly, hesperidin followed a different trend to other flavonoids, with EMAE yielding the highest concentration (36.69 mg/g d.e.), followed by EUAE (18.16 mg/g d.e.), and EUMAE (6.92 mg/g d.e.) obtaining the lowest yield.

The detailed quantification of phenolic compounds recovered was also carried out in the concentrated extracts obtained after resin purification (Figure 3). This analysis aims to assess the differences in purification efficiency for specific phenolic compounds and determine whether the purification step selectively affects their recovery.

Comparing the trends observed in Figure 2 and Figure 3, it is evident that the enhanced recovery of phenolic compounds in extracts obtained through the EUAE treatment is maintained even after purification. This process allows for the production of extracts with a higher concentration of phenolic compounds. The total content of chlorogenic acid isomers, caffeic acid, and luteolin rutinoside has doubled in most cases compared to the initial concentrations found in the crude extracts (Figure 2).

In the case of coumaric acid isomers, the purified extract derived from the EUAE treatment exhibited a fourfold increase in concentration, whereas the EMAE-PE and EUMAE-PE extracts showed a threefold increase, a trend also observed for apigenin glucoside. Notably, the flavonoid derivative luteolin rutinoside represents a substantial proportion of the total phenolic content in the EUAE-PE extract, constituting 34.9% of the total phenolic fraction. No significant differences were observed in the recovery of luteolin rutinoside between the EMAE-PE and EUMAE-PE extracts.

Following the purification process, the final concentration of phenolic compounds in the extracts (obtained by the sum of the independent phenolic compounds quantified) was as follows: 570.89 mg PC/g d.e for EMAE-PE extract, 704.68 mg PC/g d.e for EUAE-PE extract, and 454.54 mg PC/g d.e for EUMAE-PE extract. These findings confirm that resin-based purification is an effective strategy for concentrating and purifying phenolic compounds, as demonstrated by the results presented in Section 3.2.

### 3.4. Determination of Antioxidant Activity

The antioxidant capacity of the extracts was evaluated using four complementary assays: Folin–Ciocalteu, ABTS, DPPH, and FRAP, all expressed in Trolox equivalents (µmol/g d.e.) (Table 4). Although the Folin–Ciocalteu (FC) assay is commonly used for the quantification of total phenolics, it is well known that this method is not highly selective, as other antioxidant molecules can also react with the Folin reagent, potentially leading to an overestimation of the results. For this reason, in recent years, the FC assay has also been employed to assess the total antioxidant activity, serving as a complementary method alongside other in vitro antioxidant activity assays [5,66].

A one-way ANOVA was conducted for each extract across the four assays, revealing significant variations in antioxidant activity depending on the extraction methodology and the purification process. However, a general trend was observed, with the extracts obtained via EUAE and, consequently, EUAE-PE presenting the highest antioxidant capacity.

The results (Table 4) indicate significant differences in the antioxidant activity among all treatments and a marked enhancement of antioxidant activity following the purification step (PE), regardless of the extraction method used.

Regarding FC assay, the EUAE extract exhibited the highest antioxidant power (985 µmol GAE/g d.e.), followed by EUMAE (855 µmol GAE/g d.e.) and EMAE (815 µmol GAE/g d.e.). Notably, the purification process led to a threefold increase in antioxidant activity, yielding extracts with values ranging from 2643 to 2981 µmol GAE/g d.e. A similar trend was observed in the DPPH assay, where EUAE extracts demonstrated the highest antioxidant capacity (87 µmol ET/g d.e.). Purification further enhanced the antioxidant activity, resulting in a sevenfold increase for EMAE extracts and an over eightfold increase for EUAE extracts. The FRAP results follow the same trend; however, no significant differences were observed between the antioxidant activity of EUAE and EUMAE extracts. The extract with the lowest FRAP antioxidant activity was EMAE. Through resin purification, the antioxidant activity increased more than threefold for the EMAE-PE extract and approximately fourfold for the EUAE-PE and EUMAE-PE extracts.

Regarding the ABTS assay, the highest antioxidant activity was recorded for EMAE extracts (91 µmol ET/g d.e.), followed by EUAE (80 µmol ET/g d.e) while EUMAE exhibited the lowest values (64 µmol ET/g d.e). Purification with resins significantly increased antioxidant activity, with EMAE-PE showing a nearly fivefold increase (447 µmol ET/g d.e), while EUAE-PE and EUMAE-PE achieved over a sevenfold increase (593 µmol ET/g d.e and 497 µmol ET/g d.e., respectively). All results suggest that the most effective extraction method among those tested in this study is EUAE.

A comparison of the antioxidant activity results with previously published studies confirms that, as previously discussed, the antioxidant content and activity of extracts are primarily influenced by both the biological characteristics of the plant and the extraction methodology. Mena-García et al. reported higher DPPH antioxidant activity values in MAE extracts from artichoke by-products, reaching 34.48 mg (138 µmol) ET/g d.w. for artichoke leaves and 32.54 mg (128 µmol) TE/g d.w. using MAE [14]. Similarly, Laghezza-Masci et al. obtained artichoke leaf extracts with DPPH values ranging from 27.58 to 1102 µmol Trolox/g d.e., while stem extracts ranged from 462.18 to 1686 µmol Trolox/g d.e. across different artichoke genotypes. For FRAP activity, values between 114 and 243 µmol TE/g d.e. were reported for leaf extracts, while stems exhibited values ranging from 211 to 520 µmol TE/g d.e. [5].

Numerous studies have evaluated the antioxidant activity of artichoke extracts obtained from different plant parts, genotypes, and extraction methods. The high variability in the results highlights the significant influence of the artichoke variety on the final antioxidant activity of the extracts [1,4,22,55,67].

On the other hand, although all extraction methodologies are technically feasible at the laboratory scale, certain practices, such as performing multiple extraction cycles on the same matrix to deplete its antioxidant content, are not viable at an industrial level. The increased costs associated with solvent and energy consumption often render these methods economically unfeasible. However, such practices are commonly employed when evaluating the bioactivity of an extract or when aiming to reference results to the initial plant matrix, as in these cases, extracting the total bioactive compound content is of interest.

In this study, extraction methodologies with potential applicability in the food industry were assessed, including direct extraction using EUAE, EUMAE, and EMAE, which yielded extracts with high antioxidant content and activity. Additionally, three extracts obtained through concentration/purification using resins were analyzed, showing lower extraction yields but significantly enhanced antioxidant activity. These purified extracts may be particularly valuable for pharmaceutical or cosmetic applications. To determine the real feasibility of these extracts, further research is needed, not only in terms of antioxidant activity (evaluating their effects on cellular models) but also regarding their antimicrobial, anti-inflammatory, or anticancer potential, as previous studies have demonstrated for artichoke extracts.

## 4. Conclusions

This study demonstrates the feasibility and efficiency of applying green extraction technologies at a pilot scale for the recovery of phenolic compounds from artichoke by-products. Among the tested methods, the combination of enzymatic pretreatment and ultrasound-assisted extraction (EUAE) emerged as the most promising strategy, allowing for a high recovery of bioactive compounds. The phenolic profile of the extracts obtained through EUAE was particularly rich in chlorogenic acid, caffeic acid, and luteolin rutinoside, which are compounds associated with well-documented antioxidant and therapeutic effects.

Furthermore, the application of a resin-based purification step using Diaion^®^ HP20 enhanced the antioxidant potential and purity of the extracts. Notably, the EUAE-purified extract (EUAE-PE) reached the highest phenolic purity and antioxidant activity across all evaluated conditions, highlighting the value of coupling ultrasound technology with selective resin adsorption. These results suggest that EUAE, followed by purification, can yield phenolic-rich fractions with potential applications in the pharmaceutical, nutraceutical, and cosmetic sectors, where high antioxidant capacity and compound specificity are critical.

Moreover, this work represents the first pilot-scale evaluation of a hybrid ultrasound–microwave extraction system (EUMAE) for artichoke biomass, providing novel insights into the technical performance and compound selectivity of this emerging approach. Although EUMAE showed the highest extraction yield in terms of total dry mass, it did not significantly outperform EUAE in terms of phenolic concentration, indicating that extract quality should be prioritized alongside quantity in the context of high-value application.

Moving forward, further research is needed to optimize these extraction protocols for industrial implementation. This includes scaling the process to larger volumes, improving energy efficiency, and evaluating economic feasibility. Moreover, future studies should assess the physicochemical stability, bioavailability, and functionality of the extracts in real product formulations. The biological activities of the purified extracts—beyond antioxidant capacity—should also be investigated, particularly their anti-inflammatory, antimicrobial, and anticancer effects, using cellular and in vivo models.

## Figures and Tables

**Table 1:** Extraction yields, as g of extract per 100 g of dry artichoke, TPC as μmol GAE per g of dry artichoke, and TPC as μmol GAE per g of dry extract (±SD) (n = 3).

Treatment	Extraction Yield, % (m/m)	TPC μmol GAE/g d.w.	TPC μmol GAE/g d.e.
EAE	14.87 ± 0.92	44.82 ± 0.44	301.40 ± 2.07

**Table 2:** Extraction yields, as g of extract per 100 g of dry artichoke and TPC as mmol GAE per g of dry artichoke (±SD) (n = 3). Results with different letters are significantly different (*p* < 0.05).

Treatment	Extraction Yield, % (m/m)	TPC μmol GAE/g d.w.
EMAE	22.13 ± 1.34 ^a^	180.09 ± 1.94 ^a^
EUAE	21.38 ± 0.95 ^a^	210.76 ± 1.40 ^b^
EUMAE	24.68 ± 2.74 ^b^	211.35 ± 3.12 ^b^

**Table 3:** Characterization of phenolic compounds in artichoke by-product extracts obtained by EUAE, EMAE, EUMAE, EUAE-PE, EMAE-PE, and EUMAE-PE using HPLC-MS.

Peak	Rt (min)	*m*/*z* exp.	*m*/*z* calc.	Molecular Formula	Error (ppm)	Compound
Phenolic acids and derivatives						
4	2.78	353.0858	353.0878	C_16_H_18_O_9_	5.31	Chlorogenic acid isomer a
5	5.214	163.0396	163.0401	C_9_H_8_O_3_	2.58	Coumaric acid isomer a
6	5.214	179.0344	179.035	C_9_H_8_O_4_	3.06	Caffeic acid
7	6.017	353.0855	353.0878	C_16_H_18_O_9_	6.03	Chlorogenic acid isomer b
8	6.386	353.0849	353.0878	C_16_H_18_O_9_	8.21	Chlorogenic acid isomer c
9	7.203	163.0396	163.0401	C_9_H_8_O_3_	3.03	Coumaric acid isomer b
10	8.714	367.1014	367.1915	C_17_H_20_O_9_	−0.43	Feruloylquinic acid
13	12.223	515.115	515.1195	C_25_H_24_O_12_	9.31	Cynarin
Flavonoids and derivatives						
6	5.81	431.1888	431.0984	C_21_H_20_O_10_	−6.21	Apigenin glucoside
11	10.815	593.146	593.1512	C_27_H_30_O_15_	10.45	Luteolin rutinoside
12	11.763	609.1824	609.1825	C_28_H_34_O_15_	0.1	Hesperidin
Other compounds						
1	0.525	191.0573	191.0561	C_7_H_12_O_6_	−5.99	Quinic acid
2	0.835	164.0712	164.0717	C_9_H_11_NO_2_	2.84	Phenylalanine
3	1.983	203.0819	203.0829	C_11_H_12_N_2_O_2_	3.32	Tryptophan

**Table 4:** FC, ABTS, DPPH, and FRAP values of artichoke by-product extracts obtained by EMAE, EUAE, EUMAE, and the concentrated extracts EMAE-PE, EUAE-PE, and EUMAE-PE. Different letters indicate statistically significant differences at *p* ≤ 0.05.

	FC μmol GAE/g d.e.	ABTS, μmol ET/g d.e	DPPH, μmol ET/g d.e.	FRAP, μmol ET/g d.e.
EMAE	814.93 ± 6.35 ^a^	91.29 ± 1.27 ^a^	75.68 ± 0.32 ^a^	178.67 ± 3.56 ^a^
EUAE	985.33 ± 4.46 ^b^	80.46 ± 2.39 ^b^	87.03 ± 1.11 ^b^	184.99 ± 2.52 ^b^
EUMAE	855.14 ± 5.93 ^c^	63.81 ± 0.97 ^c^	45.93 ± 0.52 ^c^	186.87 ± 0.52 ^b^
EMAE-PE	2642.91 ± 10.80 ^d^	446.69 ± 6.79 ^d^	531.32 ± 5.76 ^d^	595.39 ± 4.51 ^c^
EUAE-PE	2981.35 ± 12.16 ^e^	592.64 ± 5.96 ^e^	738.31 ± 6.78 ^e^	672.41 ± 2.68 ^d^
EUMAE-PE	2688.20 ± 12.54 ^f^	497.02 ± 10.22 ^f^	391.93 ± 5.87 ^f^	649.65 ± 4.21 ^e^

## Data Availability

Data is contained within the article.

## Abbreviations

The following abbreviations are used in this manuscript:

EMAE	Enzyme–microwave-assisted extraction
EAE	Enzyme-assisted extraction
EUAE	Enzyme–ultrasound-assisted extraction
EUMAE	Enzyme–ultrasound–microwave-assisted extraction
F-C	Folin–Ciocalteu
ABTS	2,2′-azino-bis(3-ethylbenzothiazoline-6-sulfonic acid
DPPH	2,2-diphenyl-1-picrylhydrazyl
FRAP	Ferric reducing antioxidant power
HPLC	High pressure liquid chromatography
GAE	Gallic acid equivalents
TE	Trolox equivalents
PE	Purified extract
TPC	Total phenolic content
MS	Mass spectrometer
QTOF	Quadrupole time of flight
ESI	Electrospray ionization
TPTZ	2,4,6-Tris(2-pyridyl)-s-triazine
Rt	Retention time
SD	Standard deviation
Rpm	Revolutions per minute
d.w.	Dry artichoke waste weight
d.e.	Dry artichoke waste extract
S/L	Solid to liquid
